# A pipeline contributes to efficient identification of salivary proteins in short-headed planthopper, *Epeurysa nawaii*

**DOI:** 10.1038/s41598-024-56896-4

**Published:** 2024-03-14

**Authors:** Xiao-Jing Wang, Qiao Li, Zhuang-Xin Ye, Hai-Jian Huang

**Affiliations:** 1 State Key Laboratory for Managing Biotic and Chemical Threats to the Quality and Safety of Agro-products, Institute of Plant Virology, Ningbo University, Ningbo, China; 2 Animal and Plant Quarantine Service, Technology Center of Wuhan Customs District, Wuhan, China

**Keywords:** Evolution, Physiology

## Abstract

Saliva, an oral secretion primarily originating from salivary glands (SGs), exert critical roles in the ongoing evolutionary interaction between insects and plants. However, identifying insect salivary components poses challenges due to the tiny size of insects, low secretion amounts, and the propensity for degradation after secretion. In this study, we developed a transcriptome-based approach to comprehensively analyze the salivary proteins of the short-headed planthopper, *Epeurysa nawaii*, a species with unique feeding habits on bamboo. A total of 165 salivary proteins were identified, with 114 secretory genes highly and specifically expressed in SGs. Consistent with most phloem-feeding insects, digestive enzymes, calcium-binding proteins, oxidoreductases, and a few previously reported salivary effectors were ubiquitously distributed in *E. nawaii* saliva. However, we also identified a substantial portion of salivary proteins exhibiting taxonomy specificity, including 60 *E. nawaii*-specific and 62 Delphacidae-specific proteins. These taxonomy-restricted proteins potentially play a role in insect adaptation to specific host plants. Our study provides an efficient pipeline for salivary protein identification and serves as a valuable resource for the functional characterization of effectors.

## Introduction

Saliva, an oral secretion primarily originating from salivary glands (SGs), plays crucial roles in the intricate interactions between insects and their host plants. It is mainly comprised of bioactive proteins with diverse functions, ranging from overcoming plant defenses to facilitating nutrient digestion, enabling insects to feed and thrive successfully^1,2^. For example, salivary sheath protein salivary-4 from *Nilaparvata lugens* enabled insect stylet anchored in a fixed point and sealed wounded plant tissues^3^; salivary protein Bt56 from *Bemisia tabaci* suppress plant defense by leveraging SA-JA crosstalk^4^; salivary proteases from *Acyrthosiphon pisum* are capable of degrading sieve-tube proteins^5^. After secretion, the insect saliva directly contacts with plant tissues, and intimates associated with plant physiology. A few salivary proteins have been reported to experience a high evolutionary rate^6,7^. Noteworthily, some of proteins even exhibit taxonomy/species-specify, which can be only found in specific taxa^7^. Understanding the insect salivary proteins will provide a wealth of information about the complex co-evolving arms race between insects and plants, and might be useful in pest management.

In recent decades, with the development of sequencing technology, the salivary proteins of several herbivorous insects have been unveiled. Generally, insect saliva can be identified using two methods: direct analysis of secreted saliva by a proteomic approach and indirect analysis of SG extracts by a transcriptomic approach^8–11^. Although the former method is expected to provide an accurate representation of the proteins that are secreted into artificial diets, some important salivary proteins might be omitted^12^. For example, the LsSP1 protein, which is validated to be secreted and exerts essential roles in insect feeding, fails to be detected in secreted saliva using proteomic analysis^13^. Similarly, the salivary protein Bt56 can be predicated by transcriptomic analysis but not by proteomic analysis^4,14^. In proteomic analysis, trypsin is usually used for protein digestion, exclusively cleaving peptide chains at the carboxyl side of the amino acid lysine or arginine^15^. Therefore, proteins with fewer lysine or arginine residues might have been omitted using this method. Additionally, insect salivary proteins are usually of short amino acid length, which might have a lower chance of being detected by the proteomic approach^16^. When analyzing the salivary proteins that were validated to be secreted, a few conserved properties were discovered, including the secretory property represented by a signal peptide at the N-terminus and the tissue-specific property represented by high and specific expression in the SG^17,18^. These properties contribute to the efficient and accurate identification of salivary proteins.

The Delphacidae, commonly referred to as planthoppers, encompass over 2100 described species worldwide (https://sites.udel.edu/planthoppers, accessed on December 2023). Most species within this family feed on gramineous plants, with some posing significant threats as agricultural pests. Notably, the brown planthopper (*N. lugens*, BPH), small brown planthopper (*Laodelphax striatellus*, SBPH), and white-backed planthopper (*Sogatella furcifera*, WBPH) are well-known Delphacidae species, causing substantial economic damage to rice production in Asia^19^. Presently, our understanding of Delphacidae is primarily limited to these three rice planthoppers, while other planthopper species, despite their considerable size, remain poorly investigated. For instance, with the exception of the three rice planthoppers, scant transcriptomic data is available for other planthopper species in NCBI SRA database.

**Figure 1:**
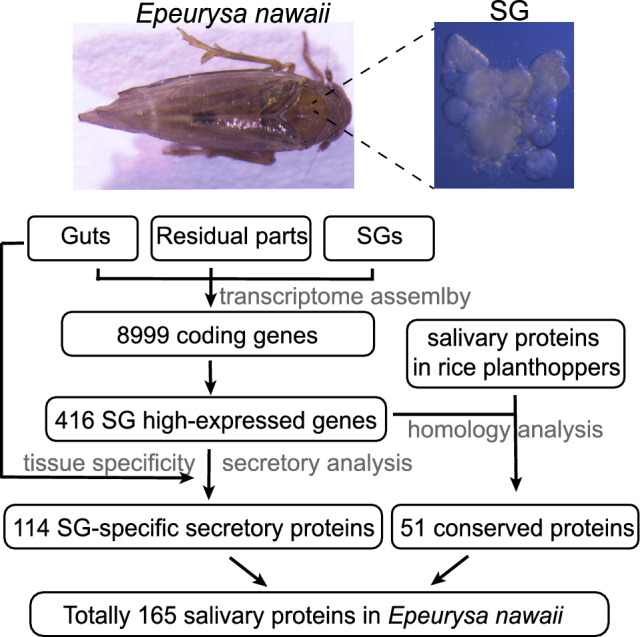
Pipeline used in analyzing salivary proteins in *Epeurysa nawaii*. Transcriptomic sequencing was performed in salivary glands (SGs), gut, and the residual parts excluding SGs and guts. The output reads were combined for Trinity assembly. Genes with an FPKM value > 100 in the SGs were defined as SG high expressed genes. Two gene categories were classified as salivary proteins: (1) secretory genes that were highly and specifically expressed in SGs; (2) SG high-expressed genes that have homology in saliva of related insect species. Totally 165 salivary proteins were identified following this pipeline.

The short-headed planthopper, *Epeurysa nawaii* Matsumura, is widely distributed in Asia. In contrast to the three rice planthoppers, which predominantly feed on grass plants, *E. nawaii* primarily feeds on and damage to bamboo^20^. It is interesting to compare salivary proteins between insects with distinct feeding habits. In this study, we conducted a comprehensive analysis of the salivary proteins of *E. nawaii* (Fig. 1). Furthermore, we compared the salivary proteins of *E. nawaii* with those of the other three planthoppers and other herbivorous insects, revealing both shared and unique saliva compounds.

## Material and methods

### Sample preparation and transcriptomic sequencing

*E. nawaii* used in this study were gathered from a bamboo grove at Ningbo University (Ningbo, China) in May 2023. Before dissection, the insects underwent a 5 s anesthetization in CO_2_ and were subsequently transferred onto ice. Insect SGs and guts were dissected in a phosphate-buffered saline (PBS) solution (137 mM NaCl, 2.68 mM KCl, 8.1 mM Na_2_HPO_4_ and 1.47 mM KH_2_PO_4_ at pH7.4) using a pair of forceps (Ideal-Tek, Switzerland) under a SZ2-ILST Stereomicroscope (Olympus, Shinjuku, Japan). Twenty insects, including male adults and female adults, were used for tissue dissection.

The isolated SGs, guts, and residual parts were homogenized in RNAiso plus (TaKaRa, Dalian, China) using FastPrep-24 5G (MP Biomedicals, Santa Ana, California, USA). The residual part sample was collected by carefully removing SGs and guts in the PBS solution under a SZ2-ILST Stereomicroscope. Twenty SGs, twenty guts, and ten residual parts were used, respectively. Total RNA extraction followed the manufacturer's protocols, and the RNA samples were forwarded to Novogene Institute (Novogene, Beijing, China) for transcriptomic sequencing. Briefly, mRNA isolation from total RNA employed poly-T oligo-attached magnetic beads. mRNA fragmentation occurred under elevated temperature using divalent cations in NEBNext First Strand Synthesis Reaction Buffer (5X). First strand cDNA synthesis utilized a random hexamer primer and M-MuLV Reverse Transcriptase. Subsequently, the second strand cDNA synthesis was carried out using DNA Polymerase I and RNase H. Blunt ends were generated by converting the remaining overhangs using exonuclease/polymerase activities. Following adenylation of DNA fragment 3' ends, NEBNext Adaptors with hairpin loop structures were ligated to prepare fragments for hybridization. To select cDNA fragments within the preferred length of 250–300 bp, library fragments were purified using the AMPure XP system (Beckman Coulter, Beverly, USA). Subsequently, 3 µl of USER Enzyme (NEB, USA) treated the size-selected, adaptor-ligated cDNA, incubating at 37 °C for 15 min, followed by 5 min at 95 °C, before PCR amplification. PCR employed Phusion High-Fidelity DNA polymerase, Universal PCR primers, and Index Primer. Finally, PCR products were purified using the AMPure XP system, and library quality was assessed using the Agilent Bioanalyzer 2100 system.

The index-coded samples were clustered using the acBot Cluster Generation System, with the TruSeq PE Cluster Kit v3-cBot-HS (Illumina), following the manufacturer’s guidelines. After cluster generation, library preparations underwent sequencing on an Illumina Novaseq6000 platform, producing 150 bp paired-end reads. One library was construct for each sample, and no biological replicate was performed. The output data were submitted to the National Genomics Data Center under accession number: PRJCA022401.

### Transcriptome assembly

Clean reads were obtained using the internal software Fastp version 0.23.1^21^, which involved the removal of low-quality reads containing adapters, empty reads, or sequences with unknown bases “N” from the raw data. The quality of the reads was assessed using the FastQC program v0.11.3^22^.

For transcriptome assembly, the left files (read1 files) from all samples were consolidated into a single large left.fq file, and the right files (read2 files) were consolidated into a single large right.fq file. Transcriptome assembly was then conducted based on the left.fq and right.fq files using Trinity^23^. The min_kmer_cov parameter was set to 2 by default, and all other parameters were set to their default values.

### Tissue-specific expressions analysis

Clean reads obtained from transcriptomic sequencing were aligned to the assembled unigenes using RNA-Seq by Expectation Maximization (RSEM)^24^. The quantification of relative transcript levels was performed using the expected number of Fragments Per Kilobase of transcript sequence per Million base pairs sequenced (FPKM), providing an accurate representation of transcript abundance. As this study lacked biological replicates, the read counts underwent additional normalization using the Trimmed Mean of M values method^25^. Differentially expressed unigenes in the SGs_vs_guts and SGs_vs_residue comparisons were identified based on predefined criteria: a fold change ratio greater than 10 and a q-value lower than 0.005^14^. The SG -high expressed genes were underwent Kyoto Encyclopedia of Genes and Genomes (KEGG) pathway enrichment analysis using TBtools software v1.069770^26^.

### Gene annotation and protein prediction

The assembled unigenes underwent analysis through the BLASTX algorithm by querying the GenBank databases. Gene Orthology (GO) and KEGG Orthology (KO) annotations for these unigenes were established using Blast2GO (http://www.blast2go.org/) and InterProScan software (http://www.ebi.ac.uk/Tools/pfa/iprscan/), respectively. Additionally, the unigenes were cross-referenced with the Clusters of Orthologous Groups (COG) database (http://www.ncbi.nlm.nih.gov/COG/).

The coding sequence of each gene was analyzed by searching against the NCBI NR, SwissProt, COG, and KEGG databases. Subsequently, the remaining unigenes underwent further prediction using TransDecoder^27^.

### Analysis of secretory proteins

The prediction of signal peptides was conducted using the SignalP 6.0 Server (https://services.healthtech.dtu.dk/services/SignalP-6.0/). For transmembrane domain prediction, each amino acid sequence containing a signal peptide was submitted to the TMHMM Server v. 2.0 (http://www.cbs.dtu.dk/services/TMHMM/). Proteins lacking transmembrane domains or having only one transmembrane domain, which partially overlapped with the predicted signal peptide, were classified as secreted proteins.

### Identification of taxonomy-specific proteins

To identify potential Delphacidae-specific proteins, the salivary proteins were subject to BLAST search against the predicted proteins in *Drosophila melanogaster*^28^, *Acyrthosiphon pisum*^29^, *Bemisia tabaci*^30^, and *Riptortus pedestris*^31^, employing a cutoff E-value of 10^–10^, respectively. Only genes with no homologous gene in above species were defined as the Delphacidae-specific genes. To identify potential *E. nawaii*-specific proteins, the Delphacidae-specific proteins were subject to BLAST search against the predicted proteins in *N. lugens*^32^, *L. striatellus*^33^, and *S. furcifera*^34^ with a cutoff E-value of 10^–10^, respectively. Only genes with no homologous gene in three rice planthopper species were defined as the *E. nawaii*-specific genes.

### Comparative analysis of insect saliva proteins

The salivary proteins from aphids (*Acyrthosiphon pisum*, *Macrosiphum euphorbiae*, *Myzus persicae*, *Diuraphis noxia*, *Schizaphis graminum*, and *Megoura viciae*)^35–40^, planthoppers (*Nilaparvata lugens*, *Laodelphax striatellus*, *Sogatella furcifera*)^18,41,42^, whiteflies (*B. tabaci*)^14^, and psyllid (*D. citri*)^11^ were extracted following published sequence information. The identification of both shared and species-specific salivary proteins among different arthropod species was accomplished by BLASTing *E. nawaii* salivary proteins against above sequences with a cutoff E-value of 10^−10^.

## Results

### Overview of *E. nawaii* transcriptome

To depict the gene expression patterns across various tissues in *E. nawaii*, cDNA libraries were prepared for SGs, guts, and residual parts excluding SGs and guts. High-throughput sequencing of these samples generated approximately 45 million paired-end (PE) reads per library. Raw reads underwent preprocessing steps, including sequence duplication removal and GC content analysis using FastQC software (Table 1). The summaries of the total clean/raw reads, the total number of bases in the clean/raw data, and the error rate of average base sequencing, Q20, and Q30 are presented in Table 1.

**Table 1:** Summary of statistics from Illumina sequencing.

Sample	Raw reads	Clean reads	Raw base (G)^a^	Clean base (G)^b^	Error (%)^c^	Q20 (%)^d^	Q30 (%)^e^	GC content (%)
SG	50,569,634	49,798,772	7.59	7.47	0.03	97.70	93.60	45.89
Gut	46,676,596	45,777,776	7.00	6.87	0.03	97.84	93.72	42.27
Residue	43,216,632	42,265,910	6.48	6.34	0.03	97.47	93.20	41.40

^a^Raw Base, The total number of bases in the raw data.

^b^Clean Base, The total number of bases in the clean data.

^c^Error, Error rate of average base sequencing.

^d^Q20, Percentage of bases with Phred quality scores > 20.

^e^Q30, Percentage of bases with Phred quality scores > 30.

Due to the absence of an available *E. nawaii* genome, the output reads were combined for Trinity assembly, resulting in a total of 18,395 unigenes with a mean length of 1678 bp and an N50 of 2200 bp (Table S1). Subsequently, initial coding sequence predictions were performed by searching against the NCBI NR, SwissProt, COG, and KEGG databases, yielding significant BLASTX hits for 11,478 unigenes. Further predictions were made using TransDecoder, identifying an additional 4304 putative proteins from the remaining unigenes. In total, our analysis predicted 15,782 coding genes in the transcriptome assembly (Table S2), providing a foundational basis for subsequent analyses.

### Functional analysis of high-expressed genes in SGs

We conducted an in-depth analysis of highly expressed genes in *E. nawaii* SGs to elucidate their primary functions. Genes with an FPKM value exceeding 100 in SGs were considered highly expressed. A total of 416 genes met this criterion (Table S3). Notably, a significant proportion of these genes were ribosomal proteins, which mainly associated with protein synthesis. Additionally, SGs exhibited a notable presence of functionally unknown or hypothetical proteins, hinting at potential associations with the rapid evolution of salivary genes. Furthermore, genes previously validated to be secreted in rice planthoppers, such as mucin-like protein, vitellogenin, annexin-like protein, and salivap-3, were highly expressed in *E. nawaii* SGs. Remarkably, over 16% of highly expressed SG genes featured a signal peptide (Table S3), suggesting their potential delivery into saliva through the eukaryotic endoplasmic reticulum–Golgi pathway.

**Figure 2:**
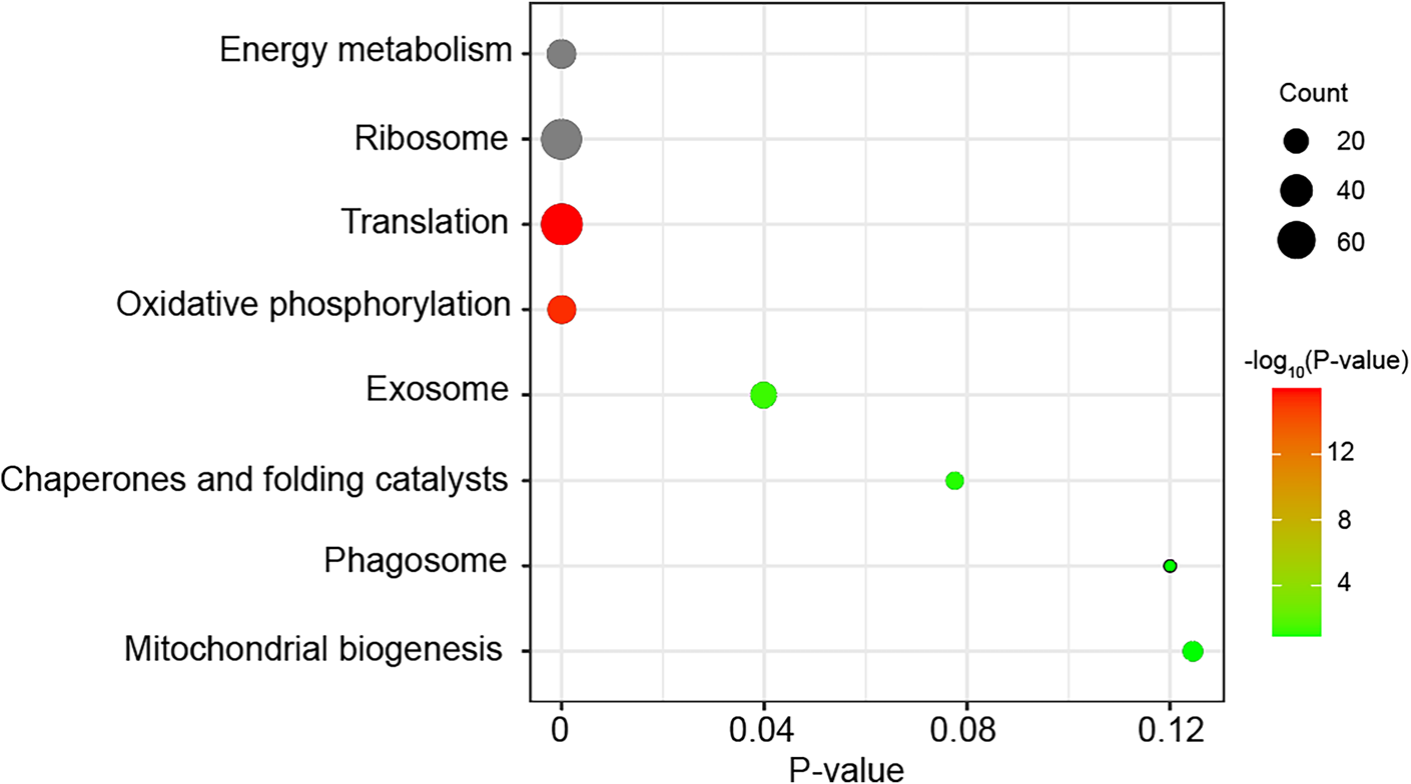
Enrichment analysis of salivary gland (SG)-high expressed genes. The SG -high expressed genes were underwent Kyoto Encyclopedia of Genes and Genomes (KEGG) pathway enrichment analysis using TBtools software^26^. Enriched *P* values were calculated according to one-sided hypergeometric test.

To gain insights into the functions of these highly expressed SG genes, KEGG pathway enrichment analysis was performed. Five pathways showed significant enrichment, including energy metabolism (31 proteins), ribosome (72 proteins), translation (76 proteins), oxidative phosphorylation (29 proteins), and exosome (23 proteins) (Fig. 2). These results suggest that SGs are primarily involved in active protein synthesis and secretion.

### Identification of salivary proteins in *E. nawaii*

Salivary proteins typically exhibit conserved characteristics, including high and specific expression in SG, the presence of a signal peptide at the N-terminus, and sequence conservation in related insect species^17,18^. In our study, we investigated the expression patterns of 416 SG-high expressed genes in SGs, guts, and residual parts. The results revealed that 180 genes displayed SG-specificity, with 43 among the Top50 high-expressed SG genes being specifically expressed in SGs (Table S3). Regarding secretory prediction, 35.1% (146 out of 416) of genes harbored a signal peptide at the N-terminus (Table S3). Notably, this proportion significantly increased in the Top50 genes, with 62% predicted to be secretable. Consequently, based on the above criteria, we identified a total of 114 secretory genes that were highly and specifically expressed in SGs (Table S3).

For the remaining 302 SG-high expressed genes, we investigated whether their homologues were present in the secreted saliva of three previously reported planthopper species. This analysis led to the identification of an additional 51 proteins, with 17 specifically expressed in SGs and 7 containing a signal peptide. Notably, the salivary effectors vitellogenin and annexin, validated for their role in impairing plant defenses^43,44^, were identified using this method, despite not being specifically expressed in SGs. Similarly, the mucin-like protein and salivap-3 protein, which did not predictably have a signal peptide, were also considered potential proteins of *E. nawaii* saliva. Collectively, we classified 165 proteins as salivary proteins of *E. nawaii*, forming the basis for subsequent analyses (Table S4).

### Comprehensive analysis of salivary proteins in different insects

According to gene annotation, 123 salivary proteins were annotated as hypothetical proteins, uncharacterized proteins, or unannotated (Table S4). To assess their species-specificity, the 165 salivary proteins were subjected to a BLAST search against protein repertoire predicated by genome in various insects, including *N. lugens*, *L. striatellus*, *S. furcifera*, *A. pisum*, *B. tabaci*, *R. pedestris*, *H. vitripennis*, and *D. melanogaster*, with a cutoff E-value of 10^–10^. The results showed that 60 salivary proteins were specific to *E. nawaii*, lacking homologues in other insects, even closely related Delphacidae species (Table S4). Additionally, 62 salivary proteins exhibited Delphacidae-specificity, with no homologues found outside the Delphacidae. In contrast, only 43 salivary proteins were prevalently existed across various insects (Table S4).

**Figure 3:**
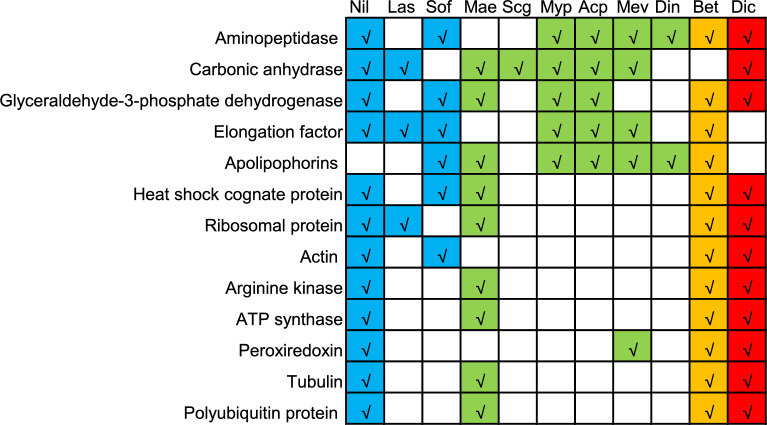
Comparative analysis of salivary proteins in *Epeurysa nawaii* with other Hemiptera species. Nil, *Nilaparvata lugens*; Lao, *Laodelphax striatellus*; Sof, *Sogatella furcifera*; Mae, *Macrosiphum euphorbiae*; Scg, *Schizaphis graminum*; Myp, *Myzus persicae*; Acp, *Acyrthosiphon pisum*; Mev, *Megoura viciae*; Din, *Diuraphis noxia*; Bet, *Bemisia tabaci*; Dic, *Diaphorina citri*. *E. nawaii* salivary proteins with their homologous in saliva of more than four species were displayed.

To date, saliva collection on artificial diets has been performed in several Hemiptera insects, including rice planthoppers (*N. lugens*, *S. furcifera*, and *L. striatellus*), aphids (*A. pisum*, *M. euphorbiae*, *M. persicae*, *D. noxia*, and *S. graminum*), psyllid (*D. citri*), and whitefly (*B. tabaci*). The salivary proteins of *E. nawaii* were compared with those of these 10 species. Seventy-nine *E. nawaii* proteins had homologues in the saliva of other insects, categorizable into 53 groups based on their gene annotation (Table S5). Seven proteins were widely distributed in insect saliva, including aminopeptidase, carbonic anhydrase, glyceraldehyde-3-phosphate dehydrogenase, elongation factor, apolipophorins, heat shock protein, and ribosomal protein (Fig. 3). Thirty proteins were exclusively found in the saliva of Delphacidae species (Table S5). For instance, the salivary sheath protein mucin and calcium-binding protein annexin were identified across all planthopper species. Salivap-5 and PI-PLC X domain-containing protein were identified in *E. nawaii*, *N. lugens*, and *S. furcifera*, but not in *L. striatellus*. Salivap-3 and salivap-4 were exclusively identified in *E. nawaii* and *N. lugens* (Table S5).

## Discussion

In this study, we conducted a comprehensive analysis of the salivary proteins of *E. nawaii* using a transcriptome-based pipeline. Our focus was on secretory proteins that were highly and specifically expressed in SG, as well as SG-high expressed proteins that had homologues in the saliva of other planthopper species. A total of 165 proteins were identified as salivary proteins, with the majority exhibiting Delphacidae-specificity, and some being specific to *E. nawaii*. Our findings present an efficient pipeline for the identification of salivary proteins, establishing a robust foundation for understanding the mechanisms of host manipulation by salivary effectors.

Saliva plays critical roles in digestion, detoxification, and manipulation of plant defenses, attracting increasing attention in recent years. However, identifying insect salivary proteins poses challenges due to the tiny size of insects, low secretion amounts, and the propensity for degradation after secretion^12^. In most cases, insect saliva is identified by directly analyzing secreted saliva in artificial diets through a proteomic approach or indirectly analyzing SG extracts via a transcriptomic approach^8–11^. In proteomic analyses, while salivary proteins have been revealed in several insect species, the number of identified salivary proteins varies greatly, ranging from less than ten in *Metopolophium dirhodum* to more than two hundred in *D. citri*^11,45^. This variability may be attributed to differences in saliva collection procedures and the sensitivity of mass spectrometry analysis. In our study, utilizing transcriptomic sequencing and bioinformatic analysis, we successfully identified the primary functions of SG and the potential salivary proteins it secretes (Fig. 1). Core salivary proteins, including digestive enzymes (aminopeptidase, cathepsin, and venom protease), calcium-binding proteins (annexin and EF-hand domain-containing protein), oxidoreductases (peroxiredoxin and glyceraldehyde-3-phosphate dehydrogenase), and reported salivary effectors (vitellogenin and carbonic anhydrase), were identified^44,46–48^. These results affirm the efficiency and reliability of our pipeline in identification of salivary proteins.

According to previous research, the majority of salivary proteins are typically secretable and specifically expressed in SG^18^. Our analysis identified 114 proteins that met these criteria. However, a few proteins were highly and specifically expressed in SG without a detectable signal peptide. This discrepancy may be attributed to partial sequence assembly or inaccurate protein prediction. Additionally, unconventional secretion pathways and limitations in signal peptide-based predictions could contribute to the absence of signal peptides in some salivary proteins^49^. Although most salivary proteins displayed SG-specificity, exceptions were noted in some salivary effectors. For instance, salivary maltase and trypsin-like protease, involved in extra-oral digestion, were specifically expressed in guts^18,50^. Vitellogenin, a salivary effector suppressing plant defenses, was mainly expressed in ovaries and fat bodies^44,48^. While our study employed homology analysis to include conserved proteins found in saliva of closely related species, further investigation is warranted to capture potentially omitted salivary proteins.

The intriguing feature of *E. nawaii* saliva lies in the abundance of taxonomy-specific proteins, known as orphan proteins. Saliva plays a crucial role in the evolutionary battle between plants and herbivorous insects^1,2^. Orphan salivary genes have been reported as critical for insect survival, such as Bsp9 in whiteflies for suppressing plant defenses^51^, salivap-4 in planthoppers for salivary sheath formation^3^, and C002 in aphids foraging and feeding^52^. In *E. nawaii*, 60 salivary proteins exhibited *E. nawaii*-specificity, with no homologues found even in closely related Delphacidae species (Table S4). Given the distinct feeding habits of *E. nawaii* compared to *N. lugens*, *S. furcifera*, and *L. striatellus*, which primarily feed on rice plants, our findings suggest that feeding habits may influence the composition of salivary proteins. The origin of species-specific proteins is a crucial aspect of insect evolution^53^. While previous research demonstrated horizontal gene transfer as a mechanism for recruiting orphan salivary proteins^6,54^, no homologues of *E. nawaii*-specific proteins were found in other organisms outside the Insecta, warranting further research to uncover their origin and function.

In conclusion, our study provided an efficient and reliable pipeline for identifying salivary proteins. The widespread identification of digestive enzymes, calcium-binding proteins, and oxidoreductases indicate the conserved role of saliva in mediating plant–insect interactions. The abundant presence of orphan salivary proteins underscores the importance of these proteins for insects to adapt to specific host plants.

### Supplementary Information

Supplementary Information 1.

Supplementary Information 2.

Supplementary Information 3.

Supplementary Information 4.

Supplementary Information 5.

## Data Availability

The output transcriptomic data were submitted to the National Genomics Data Center under accession number: PRJCA022401.
