# Author Correction: A pipeline contributes to efficient identification of salivary proteins in short-headed planthopper, *Epeurysa nawaii*

**DOI:** 10.1038/s41598-024-57911-4

**Published:** 2024-03-27

**Authors:** Xiao-Jing Wang, Qiao Li, Zhuang-Xin Ye, Hai-Jian Huang

**Affiliations:** 1 State Key Laboratory for Managing Biotic and Chemical Threats to the Quality and Safety of Agro-products, Institute of Plant Virology, Ningbo University, Ningbo, China; 2 Animal and Plant Quarantine Service, Technology Center of Wuhan Customs District, Wuhan, China

Correction to: *Scientific Reports* 10.1038/s41598-024-56896-4, published online 14 March 2024

In the original version of this Article Hai-Jian Huang was incorrectly affiliated with ‘Animal and Plant Quarantine Service, Technology Center of Wuhan Customs District, Wuhan, China’. Their correct affiliation is listed below.

State Key Laboratory for Managing Biotic and Chemical Threats to the Quality and Safety of Agro-products, Institute of Plant Virology, Ningbo University, Ningbo, China.

The original Article has been corrected.

